# Cytoplasmic diversity of potato relatives preserved at Plant Breeding and Acclimatization Institute in Poland

**DOI:** 10.1007/s11033-020-05486-4

**Published:** 2020-05-13

**Authors:** Paulina Smyda-Dajmund, Jadwiga Śliwka, Marta Janiszewska, Ewa Zimnoch-Guzowska

**Affiliations:** Plant Breeding and Acclimatization Institute—National Research Institute, Młochów Research Center, Platanowa 19, 05-831 Młochów, Poland

**Keywords:** Chloroplast, Cytoplasm types, Mitochondrion, *Solanum*, Wild potato species

## Abstract

Among different types of potato cytoplasmic genomes, some are associated with male sterility or affect agronomic traits. The goal of this study was to analyze types of chloroplast and mitochondrial genomes of selected potato relatives originating from collection of the Institute of Plant Industry, Saint Petersburg, Russia, and preserved in Poland. Using chloroplast and mitochondrial markers the cytoplasm types were determined for 401 genotypes belonging to 43 seed accessions of 28 *Solanum* species. Among characterized genotypes, 201 (50.1%), 156 (38.9%) and 44 (11%) had cytoplasm types W, D, M, respectively. No accessions with the T, P or A cytoplasm were found. Within cytoplasm W, genotypes with the subtypes: W/α and W/β were identified, but not with W/γ. In *S. famatinae*, we detected unusual product of the T marker with 65 bp insertion earlier seen exclusively in *S. vernei*. Among the genotypes of *S. leptophyes*, two profiles of the ALM_4/ALM_5 marker were observed. *S. famatinae* and *S. vernei* come from Argentina, provinces Catamarca and Tucumán. Possibly the insertion in marker T occurred independently in two species, or the accessions were misidentified. Segregation of the ALM_4/ALM_5 marker within *S. leptophyes* indicates that potato seed accessions are heterogeneous not only due to nuclear DNA polymorphisms but have diversified cytoplasm, too. Our findings are important for exploitation of the tested material in potato breeding. Male-fertile cytoplasm types give a chance of avoiding fertility problems and widening the range of crosses in future generations of breeding materials.

## Introduction

Potato, *Solanum tuberosum* ssp. *tuberosum* is an autotetraploid crop, vegetatively propagated with high heterozygosity and strong inbreeding depression. It is characterized by different types of cytoplasmic genomes, among which a sterilizing cytoplasm of *S. tuberosum* invasively dominates in cultivated potato [1]. This type of cytoplasm is associated with nuclear-cytoplasmic male sterility, which invoked restrictions in sexual hybridization of potato. Today, there is a need of continuous supply of new genetic diversity of potato, because the potato breeding is facing new challenges. Characterization and selection of parents in relation to the cytoplasmic type is the key to the successful breeding programs. Potato relatives are good sources of different types of cytoplasm and due to adaptation to various, often extreme environmental conditions, they show greater tolerance to various environmental stresses and are resistant to many pathogens [2]. Wild potato species are originated from North and South America, mainly from Peru, Mexico, Bolivia, Argentina, Venezuela, Colombia and Ecuador [3].The taxonomic classification of wild potato species is complex and is the subject to constant verification. Wild and cultivated potato species belong to the genus *Solanum*, section *Petota*. The *Petota* section brings together 228 species, grouped in 21 taxonomic series [4]. This number was reduced to 203 [5], then 188 [6], currently it is assumed to be 122 [7].

Chloroplast and mitochondrial DNA are maternally inherited in most of higher plants [8]. In potato, based on the molecular markers specific for mitochondrial (mtDNA) and chloroplast (cpDNA) DNA, five types of mtDNA (α, β, γ, δ, ε) and five types of cpDNA (W, C, T, A, S) were distinguished [9]. It was noticed that a given type of mtDNA is linked with a specific type of cpDNA. Based on that, six types of cytoplasmic DNA: T/β, W/α, W/γ, W/δ, A/ε and S/ε were distinguished [9]. Hosaka and Sanetomo [10] have developed a simpler method of identifying cytoplasmic DNA. Based on six markers specific for mt- and cpDNA, they distinguished six types of cytoplasmic DNA: W (W /α, W/β, W/γ), T (T/β), D (W/α), A (A/ε), P (S/ε) and M (C/ε).

Mutual interactions of nucleus and cytoplasm (plastids/mitochondria) affect many agronomic traits in crop plants. The best known trait controlled by the nuclear and mitochondrial genomes is cytoplasmic male sterility [11]. This trait is manifested in different ways depending on the type of cytoplasm. Type T (T/β) causes cytoplasmic male sterility that manifests as lack of pollen or poor pollen shedding, deformation of anthers or pollen grains. Potato varieties, characterized by the type of cytoplasm W (W/γ), derived from *S. stoloniferum*, produce small amounts of pollen grains that do not separate in the process of microsporogenesis, forming characteristic tetrads (so called tetrad sterility) [12, 13]. Forms of type D (W/α), derived from *S. demissum*, produce the right amount, morphologically unchanged pollen, however, unable to fertile with *S. tuberosum* [13]. Type T (T/β) dominates among cultivated potato varieties, which is a consequence of using these forms as seed parents, because of pollen sterility. It was also noticed that offspring of mothers with T cytoplasm (*S. tuberosum* ssp. *tuberosum*) had higher tuber yield, higher tuber number, and earlier vine maturity in comparison with that of A cytoplasm type (*S. tuberosum* ssp. *andigena*) [14–19].

Potato species and valuable interspecific *Solanum* hybrids are maintained in many genebank collections worldwide [7]. Chimote and collaborators [20] analyzed variation of chloroplast and mitochondrial genomes in potato varieties and advanced hybrids using PCR markers specific for chloroplast [1] and mitochondrial DNA. The cytoplasm types of Japanese potato collection were determined with type specific DNA markers [10]. The same markers were used for cytoplasmic characterization of European potato cultivars and breeding clones preserved at Max-Planck Institute for Plant Breeding Research [17] and International Potato Center (CIP) potato breeding germplasm [21]. They all proved that sterilizing T-type cytoplasm of *S. tuberosum* is predominant in analyzed collections and they observed an increasing number of forms with sterilizing D and W/γ cytoplasm types. For these reasons, identification of cytoplasm types and introduction of the fertile ones into new cultivars is crucial for maintaining biodiversity of potato. Potato somatic hybrids are also a valuable source of cytoplasmic diversity. Not only segregation of cytoplasm of parental forms [22] but also changes in cp- and mtDNA have been observed among somatic hybrids [23, 24].

A fraction of wild and cultivated potato species from historical, unique collection of the Institute of Plant Industry (VIR) (Saint Petersburg, Russia) is preserved in Poland, in Plant Breeding and Acclimatization Institute—National Research Institute (IHAR-PIB) [25]. Bukasov, Voronov and Juzepczuk described 30 wild and 18 cultivated species collected in Central and South America [7, 25]. These species were the beginning of the VIR potato collection [7, 25]. True seeds of 111 accessions of wild and cultivated potato species from VIR collection are maintained at IHAR-PIB Genebank. It is a result of reproducing this collection as a part of the Cornell-Eastern Europe-Mexico Project on Late Blight Control [26].

The goal of this study was to analyze types of chloroplast and mitochondrial genomes of selected wild potato species originating from VIR potato collection. In the present study four markers specific to cpDNA and two markers specific to mtDNA were used to investigate the cytoplasmic genome types according to Hosaka and Sanetomo [10].

## Materials and methods

### Plant materials

Forty-three accessions of 28 tuber-bearing wild potato species were evaluated using a set of cytoplasmic markers. They are preserved at the Plant Breeding and Acclimatization Institute – National Research Institute (IHAR-PIB) as a part originated from VIR collection (Saint Petersburg, Russia) described earlier by Zoteyeva et al. [25]. These species have been selected in order to search for new sources of resistance to *P. infestans*. Seeds (30 seeds per accession) were sown in three years 2013, 2016 and 2017. From 20 to 30 plants per accession were obtained. In 2013 four plants per accession and in 2016 and 2017 ten plants per accession were maintained. Wild species *S. michoacanum* (W type), cv. Early Rose (T type), breeding line PW 363 (D type), cultivated species *S. phureja* (P type) and cv. Maris Piper (A type) were used as a standard for multiplex PCR analysis [10]. As a standard for α, β, γ types cv. Nevsky, Early Rose and Stobrawa were used.

### DNA extraction, PCR and restriction digestion

From 1 to 14 individual plants per accession were used for DNA extraction. Total DNA was extracted from 200 mg of fresh, young leaves of greenhouse-grown plant using DNeasy Plant Mini Kit (Qiagen, Hilden, Germany). The DNA quality was checked with a NanoDrop Lite Spectrophotometer (Thermo Scientific) and assessed on 1% agarose gels. Genomic DNA was used in multiplex polymerase chain reaction (PCR) amplification with cpDNA- and mtDNA-specific primers and digested with *Bam*HI restriction enzyme. Four chloroplast-specific markers (T, S, SAC, A) and one mitochondrial DNA marker (D) were used. MtDNA types α, ß and γ were distinguished using the ALM_4 and ALM_5 primers. To confirm the identification of M-type, an additional single PCR of marker A along with digestion was performed. Product amplification, restriction digestion and agarose gel electrophoresis were done according to Hosaka and Sanetomo [10] with modifications described in Smyda-Dajmund et al. [22]. Amplification reactions (20 µl reaction mixture) consisted of 2 µl 10 × buffer including 20 mM MgCl_2_ (Fermentas Life Sciences, Thermo Fischer Scientific, Waltham, Massachusetts, USA), 0.5 mM of each dNTP, 2 µM primer T, S and SAC and 3 µM primer D and A, 0.05 U/µl DreamTaq polymerase (Fermentas Life Sciences, Thermo Fischer Scientific, Waltham, Massachusetts, USA) and 30 ng DNA template. Amplifications were performed in a T3000 thermocycler (Biometra GmbH, Göttingen, Germany). The PCR parameters for multiplex PCR were 95 °C for 10 min followed by 35 cycles at 94 °C for 30 s, 60 °C for 30 s, 72 °C for 60 s and one final extension at 72 °C for 5 min. Digestion of the amplicons with restriction endonuclease *Bam*HI (Fermentas Life Sciences, Thermo Fischer Scientific, Waltham, Massachusetts, USA) was performed according to producer’s protocol at 37 °C for 3 h. The PCR amplification of ALM_4 and ALM_5 [13] marker were prepared in volume 20 µl using 2 µl 10 × buffer including 20 mM MgCl_2_ Fermentas Life Sciences, Thermo Fischer Scientific, Waltham, Massachusetts, USA), 3 µM of each ALM_4 and ALM_5 primers, 0.05 U/µl DreamTaq polymerase (Fermentas Life Sciences, Thermo Fischer Scientific, Waltham, Massachusetts, USA) and 30 ng DNA template. The reaction conditions were incubation at 95 °C for 10 min followed by 35 cycles at 94 °C for 30 s, 57 °C for 60 s, 72 °C for 90 s and one final extension at 72 °C for 5 min. The products of multiplex PCR were separated in 1.5% high resolution agarose gels (EURx, Gdańsk, Poland). Amplicons of ALM_4 and ALM_5 marker were separated in 1.5% standard agarose gels. PCR products were visualized by ethidium bromide staining and were assessed under UV light after electrophoresis in 1 × TBE buffer (Tris–Borate-EDTA). A 100-bp DNA ladder (Invitrogen, Thermo Fischer Scientific, Waltham, Massachusetts, USA) was used to determine marker sizes.

### Sequencing

For PCR product sequence analysis of T marker, four genotypes of *S. famatinae* (RUS001:4304; POL003:333139) were chosen. The PCR products were cut out from the agarose gel under UV light and purified with a GenElute Gel Extraction Kit (Sigma-Aldrich, St. Louis MO, USA) according to the manufacture’s protocol. DNA sequencing was done by Genomed S. A, Warsaw, Poland.

## Results

**Fig. 1:**
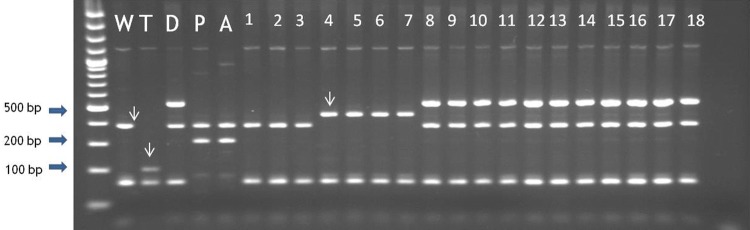
Different amplification of T marker in multiplex PCR after *Bam*HI digestion within *S. famatinae* (RUS001:4304; POL003:333139) individuals (samples 4–7). Samples 1–3 *S. dolichostigma* (RUS001:7613; POL003:333115); 8–11 *S. fendleri* (RUS001:5751; POL003:333112); 12–15 *S. fendleri* (RUS001:5747; POL003:333143); 16–18 *S. fendleri* (RUS001:5671; POL003:333110). Standards: wild species *S. michoacanum* (W), cv. Early Rose (T), breeding line PW 363 (D), cultivated species *S. phureja* (P) and cv. Maris Piper (A). T marker is marked with an arrow

Cytoplasm types were determined for 43 accessions of 28 wild potato species, in total 401 genotypes (Table 1). In the accession of *S. kurtzianum* (RUS001:2301; POL003:333130) only one plant was obtained and preserved. From 1 to 14 genotypes per accession were analyzed. Twenty-two accessions had W cytoplasmic type, 17 had D – type and 4 had M-type. No segregation of cytoplasm types was observed within individuals of the same accession. From all 401 genotypes characterized, 201 (50.1%), 156 (38.9%) and 44 (11%) had cytoplasm types W, D, M, respectively (Table 1). No accessions with the T, P or A cytoplasm types were found. Within cytoplasm W, two subtypes W/α and W/β were identified. There was no accession identified with cytoplasm type W/γ. Different than standard marker pattern was observed within 14 genotypes of *S. famatinae* (RUS001:4304; POL003:333139). In order to determine which marker amplified in multiplex PCR in different way, single PCR for T and D markers were performed. An unusual profile was obtained with the T marker (Fig. 1). The obtained product was sequenced and a sequence of 502 bp was deposited in the National Center for Biotechnology Information (NCBI) GenBank database under the MN723848 accession number. Then the sequence was used to search for sequences of greatest similarity (i.e., the lowest e value and the greatest identity and maximum coverage) within the GenBank database. The closest matching sequences were found among the complete plastid genome sequences of wild and cultivated diploid potatoes belonged to *Solanum*, section *Petota* [27]. The query sequence was identical to the *S. vernei* PI320332 (sequence ID: NC_041633.1) plastid genome region spanning a range from 52,416 to 52,917 bps. Both sequences contained 65 bp insertion that differentiated them from standard products obtained with T marker, characteristic for W cytoplasm type.

**Table 1:** Cytoplasm type of wild potato species originating from the Institute of Plant Industry, preserved in Plant Breeding and Acclimatization Institute—National Research Institute

*Solanum* species	Accession number VIR RUS001	Accession number POL POL003	Number of plants	Cytoplasm type			Marker banding pattern of ALM_4/ALM_5
S. acaule	9795	333155	10			M	3
*S. aemulans*	9146	333119	10		D		2
*S. albicans*	9814	333125	10			M	2
*S. angustisectum*	2733	333133	4	W			2
*S. antipovichii*	2354	333099	14	W			2
*S. arrac-papa*	9742	333150	14	W			2
*S. berthaultii*	23047	333129	10	W			1
*S. dolichostigma*	7610	333114	14	W			2
*S. dolichostigma*	7613	333115	4	W			2
*S. famatinae*	4304	333139	14	W^a^			2
*S. fendleri*	5751	333112	10		D		2
*S. fendleri*	5747	333143	4		D		2
*S. fendleri*	5671	333110	14		D		2
*S. gibberulosum*	2739	333134	4	W			2
*S. gibberulosum*	2937	333103	10	W			2
*S. guerreroense*	18407	333096	14		D		2
*S. hougasii*	8818	333148	14	W			2
*S. kurtzianum*	9719	333121	10	W			1
*S. kurtzianum*	2301	333130	1	W			1
*S. latisectum*	2722	333132	4	W			2
*S. leptophyes*	5764	333113	10	W^b^			1,3^b^
*S. microdontum*	9726	333149	10	W			2
*S. neoantipovichii*	8505	333117	14		D		2
*S. papita*	8816	333147	14		D		2
*S. papita*	16888	333159	14		D		2
*S. papita*	16889	333160	4		D		2
*S. papita*	17454	333161	4		D		2
*S. papita*	9145	333081	4		D		2
*S. parodii*	3701	333069	14	W			2
*S. parodii*	8280	333116	4	W			2
*S. polytrichon*	7423	333075	4		D		2
*S. polytrichon*	5347	333108	14		D		2
*S. polytrichon*	5682	333111	4		D		2
*S. polytrichon*	8815	333118	4		D		2
*S. punae*	4263	333138	14			M	3
*S. ruiz-ceballosii*	7370	333074	14	W			2
*S. ruiz-ceballosii*	7381	333144	4	W			2
*S. simplicifolium*	5400	333141	14	W			2
*S. simplicifolium*	5684	333142	4	W			2
*S. sparsipilum*	9808	333124	10	W			2
*S. stoloniferum*	2492	333100	10		D		2
*S. uyunense*	4114	333071	10			M	2
*S. verrucosum*	10556	333157	14		D		2
Total			401	201 (50.1%)	156 (38.9%)	44 (11%)	

^a^W cytoplasm type with different banding pattern type of T marker amplified in multiplex PCR

^b^ Five plants of *S. leptophyes* (RUS001:5764; POL003:333113) had mtDNA type 1 (W/β) and five were with mtDNA type 3 (W/type 3) based on ALM_4/ALM_5 marker

Analysis based on multiplex PCR (T, S, SAC, A and D markers) indicated lack of segregation of cytoplasm types among individuals belonging to the same accession (Table 1). We also observed uniform cytoplasm type for the individuals of the same species with different accessions. As an example, five accessions of *S. papita* and four accessions of *S. polytrichon* were all with D cytoplasm type (Table 1). ALM_4/ALM_5 mtDNA primers generated five (type 0, 1, 2, 3, 4) different banding patterns among standards [10]. Band patterns of types 1, 2 and 3 were the only ones present among the analyzed accessions. Types 1, 2, 3 were observed when ALM_4/ALM_5 marker was used with the potato genotypes of W cytoplasm and 2, 3 among individuals with M cytoplasm type. All of the accessions of the cytoplasm D type had type 2 band pattern of the ALM_4/ALM_5 marker. We observed segregation of ALM_4/ALM_5 marker among individuals of *S. leptophyes* (RUS001:5764; POL003:333113). Out of 10 analyzed plants, five had profile 1 and five were with type 3 (Table 1).

## Discussion

The study provides first data on cytoplasm diversity in a part of Vavilov potato collection multiplied in Poland. We have determined the types of cpDNA and mtDNA of 401 genotypes preserved in IHAR-PIB, based on a molecular marker system elaborated by Hosaka and Sanetomo [10]. We identified three cytoplasmic types: W (50.1%), D (38.9%) and M (11%) in the analyzed forms. Within type W subtypes W/α and W/β were identified. Fourteen genotypes of *S. famatinae* (RUS001:4304; POL003:333139) had W cytoplasm type, but with different amplicon of T marker. There was no sterilizing subtype W/γ. Type W and D dominated among tested genotypes and T-cytoplasm was not identified. Male-fertile cytoplasm types frequent in the material should be preserved and introduced into breeding lines in order to avoid fertility problems and widen the range of possible crosses in future generations. The dominance of a given type of cytoplasm depends on the types of materials stored in the genebank. Currently, the T type cytoplasm dominates among the potato cultivars and genebank resources. Japanese collection consists of 84 Japanese varieties and 378 breeding lines, 26 landraces and 260 foreign varieties and breeding lines [10]. The authors noticed dominance of cytoplasm T (73.9%) and D (17.4%). The frequency of cytoplasm W was slightly different than in other collections and was 2.4%. Domination of sterilizing cytoplasm types has also been noticed in other potato collections. From 978 genotypes of CIP collection 440 (45%), 368 (37.6%) and 110 (11.2%) had cytoplasm T, D and W, respectively [21]. Other analyzed genotypes had cytoplasm types: A, M and P with frequency 5.4%, 0.5% and 0.2%, respectively. The authors did not use ALM_4/ALM_5 marker and determination of sterilizing cytoplasm type W/γ was impossible, but they predicted its high frequency in CIP materials because of the intensive usage of *S. stoloniferum* as seed parent in breeding programs. 1,217 European potato cultivars and breeding clones maintained in German potato collection had T (59.4%), D (27.4%), W (12.2%) cytoplasm types. Remaining forms had A (0.7%) and M (0.3%) cytoplasm [17]. Cytoplasm types of 185 potato cultivars bred in Russia and Former Soviet Union countries were also described [28]. The authors have identified cytoplasm types: T, D and W/γ with frequency 40%, 50.8% and 8.7%, respectively. T cytoplasmic type is the most prevalent within *S. tuberosum* ssp. *tuberosum*, but it was also noticed in some forms of *S. tuberosum* ssp. *andigena* and diploid species of *S. stenotomum* [29–31]. It is not specific for wild potato species, but some accession of *S. tarijense*, *S. berthaultii* and *S. neorossii* were classified as T type [31]. Domination of T cytoplasm in European potatoes is caused by its origin from common ancestors: ‘Rough Purple Chili’, ‘Garnet Chili’ and ‘Early Rose’ and others *S. tuberosum* ssp. *tuberosum* clones [17, 32–34]. The second cytoplasm frequent in potato collections and also sterilizing is D-type. Its presence is associated with the introduction of resistance to *P. infestans* from *S. demissum* with cytoplasmic D-type in breeding programs. Cytoplasmic subtype W/γ was introduced into potato genepool from seed parents of *S. stoloniferum* carrying PVY resistance *Ry*_*sto*_ gene. This cytoplasm type was also found in *S. chacoense*, *S. pampasense*, *S. pinnatisectum* and *S. vernei* [10]. The choice of seed parent is crucial to reduce invasive sterilizing cytoplasm types.

T marker is an *ndhlC*/*trn* intergenic region in potato plastid genome [20]. The size of amplified fragment depends on potato species: approximately 440 bp fragment is specific for wild species or *S. tuberosum* ssp. *andigena*, a fragment of approximately 200 bp size is observed among *S. tuberosum* ssp. *tuberosum*, indicating a 241 bp deletion [31]. Hosaka [31] identified a longer product of T marker (of approximately 500 bp in size) among three accessions of *S. vernei*, with an insertion of 65 bp, a duplicated sequence of the position from 219 to 283 bp. We identified a PCR product of 502 bp in *S. famatinae* (RUS001:4304; POL003:333139), which was identical to the *S. vernei* PI320332 (sequence ID: NC_041633.1) plastid genome region spanning a range from 52,416 to 52,917 bps [27]. Both sequences contained 65 bp insertion that differentiated them from standard products obtained with T marker, characteristic for W cytoplasm type (437 bp in size). This was confirmed by comparing the obtained sequence with the plastid sequence of selected wild species deposited at NCBI by Huang et al. [27]. We also noticed that six *S. vernei* and two *S. spegazzinii* (synonym *S. famatinae*) accessions do not have an insertion in the T marker [27]. Both *S. famatinae* (synonym *S. spegazzinii*) and *S. vernei* come from Argentina, province Catamarca and Tucumán [4]. They are diploid wild species with EBN = 2. They belong to the same *Tuberosa* series, group Bolivia, Argentina and Chile according to Hawkes [4]. It is possible that this change occurred independently in both species, or they are the same species collected by independent collectors, misidentified and maintained in different genebanks. The argument for that hypothesis is their common geographical origin, but further analyses are necessary.

No segregation of markers T, S, SAC, A and D was observed both within the accessions and within species with multiple accessions. Hosaka and Sanetomo [10, 35] observed segregation of cytoplasm type within the same species. Lack of segregation of cytoplasm type in our material may result from the structure of VIR collection. Within *S. leptophyes* (RUS001:5764; POL003:333113), two types 1 and 3 of mitochondrial ALM_4/ALM_5 marker were observed. Segregation of this marker indicated that potato seed accessions are heterogeneous due to both nuclear and cytoplasm DNA polymorphisms. Hosaka and Sanetomo [10, 35] noticed segregation of cytoplasm types and marker ALM_4/ALM_5 within the species, but not within the same accession. The PCR reaction was carried out on DNA isolated twice from the same plants, but accidental mix of the seed material cannot be excluded.

Male-fertile cytoplasm types frequent in the material, if preserved and introduced into breeding lines, give a chance of avoiding fertility problems and widening the range of possible crosses in future generations of breeding materials.

## Data Availability

Plant material is maintained at Plant Breeding and Acclimatization Institute –National Research Institute in Poland. None.
